# No Evidence That Hyperpnea-Based Respiratory Muscle Training Affects Indexes of Cardiovascular Health in Young Healthy Adults

**DOI:** 10.3389/fphys.2020.530218

**Published:** 2020-12-17

**Authors:** Fernando G. Beltrami, David Mzee, Christina M. Spengler

**Affiliations:** ^1^Exercise Physiology Lab, Institute of Human Movement Sciences and Sport, ETH Zurich, Zurich, Switzerland; ^2^Zurich Center for Integrative Human Physiology (ZIHP), University of Zurich, Zurich, Switzerland

**Keywords:** respiratory muscle training, pulse wave velocity (PWV), blood pressure, hyperpnea, cardiovascular health, lower limb vibration

## Abstract

**Introduction:**

The chronic effects of respiratory muscle training (RMT) on the cardiovascular system remain unclear. This investigation tested to which degree a single sessions of RMT with or without added vibration, which could enhance peripheral blood flow and vascular response, or a 4-week RMT program could result in changes in pulse wave velocity (PWV), blood pressure (systolic, SBP; diastolic, DBP) and other markers of cardiovascular health.

**Methods:**

Sixteen young and healthy participants (8 m/8f) performed 15 min of either continuous normocapnic hyperpnea (RMET), sprint-interval-type hyperpnea (RMSIT) or a control session (quiet sitting). Sessions were performed once with and once without passive vibration of the lower limbs. To assess training-induced adaptations, thirty-four young and healthy participants (17 m/17f) were measured before and after 4 weeks (three weekly sessions) of RMET (*n* = 13, 30-min sessions of normocapnic hyperpnea), RMSIT [*n* = 11, 6 × 1 min (1 min break) normocapnic hyperpnea with added resistance] or placebo (*n* = 10).

**Results:**

SBP was elevated from baseline at 5 min after each RMT session, but returned to baseline levels after 15 min, whereas DBP was unchanged from baseline following RMT. Carotid-femoral PWV (PWV_CF_) was elevated at 5 and 15 min after RMT compared to baseline (main effect of time, *P* = 0.001), whereas no changes were seen for carotid-radial PWV (PWV_CR_) or the PWV_CF_/PWV_C__R_ ratio. Vibration had no effects in any of the interventions. Following the 4-week training period, no differences from the placebo group were seen for SBP (*P* = 0.686), DBP (*P* = 0.233), PWV_CF_ (*P* = 0.844), PWV_CR_ (*P* = 0.815) or the PWV_CF_/PWV_CR_ ratio (*P* = 0.389).

**Discussion/Conclusion:**

Although 15 min of RMT sessions elicited transient increases in PWV_CF_ and SBP, no changes were detected following 4 weeks of either RMET or RMSIT. Adding passive vibration of the lower limbs during RMT sessions did not provide additional value to the session with regards to vascular responses.

## Introduction

Regular, adequate physical exercise brings different adaptations to the cardiovascular system, which help mitigate the risk of cardiovascular events (Ramos et al., 2015). Endurance exercise leads to the decrease of systolic (SBP) and diastolic blood pressure (DBP) (Pattyn et al., 2013), and also to decreases in vascular stiffness, which has recently been shown to be independently associated with mortality risk (Laurent et al., 2001). The effects of exercise on the cardiovascular system can be attributed to enhanced cardiac vagal tone, improved peripheral baroreflex function as well as enhanced endothelial function (Joyner and Green, 2009). Against the backdrop of an increasingly sedentary population, in which people might choose not to engage in whole-body exercise or suffer from conditions which preclude them from exercising, there is a continuous search for alternative interventions which can deliver some of these benefits without the performance of whole-body exercise (Heffernan et al., 2007b).

One possibility is the use of respiratory muscle training (RMT) (Illi et al., 2012), in which intensive respiratory maneuvers are performed while no activity is required from the upper or lower limbs. Large decreases in systolic (SBP) and diastolic (DBP) blood pressure in normotensive adults were found after 6-weeks of strength-like RMT, but only when large pressures were used (Vranish and Bailey, 2015; DeLucia et al., 2018), a finding which has not been supported by others (Witt et al., 2007), including in more frail populations (Chiappa et al., 2008). Much less is known about potential changes on arterial stiffness brought by RMT, with a single investigation suggesting increased arterial compliance following RMT, without measuring PWV (DeLucia et al., 2018). This is a topic that needs further clarification, as for example endurance and resistance training have been shown to decrease and increase arterial stiffness respectively (Collier et al., 2008), the latter possibly due to the pressure swings resulting from Valsalva maneuvers during intense muscle contractions (Heffernan et al., 2007b). With regards to vascular stiffness, RMT also likely causes significant oscillations in the discharge from the arterial baroreceptors given the large variations in intrathoracic pressures and blood flow that can be produced (Aliverti et al., 2009), and therefore could also have an impact in log-term modulation of the sympatho-vagal balance. Indeed, 6 weeks of respiratory muscle endurance training (RMET) led to an increase in cardiac vagal tone (Hepburn et al., 2005), although this finding that was not corroborated by a longer (8-week) intervention in young healthy women (Bisconti et al., 2018).

A second intervention which has been proposed to affect vascular health is the use of vibration platforms (van Duijnhoven et al., 2010). Whole-body or lower limb vibration has been shown to acutely increase blood flow to the lower limbs and to reduce peripheral arterial resistance (Koutnik et al., 2014). However, vibration platforms are often used along with a variety of whole-body exercises, such as squatting (Figueroa et al., 2014), and the effects of passive vibration is far less well understood. Interestingly, the respiratory pump also has the potential to increase blood flow to the lower limbs during RMT (Aliverti et al., 2009) and thus the two interventions could potentially have synergistic effects when performed simultaneously, for example when performing breathing exercises while seated with the legs placed on a vibration platform.

Despite the potential for combination, there are currently too many unknowns about these two interventions, particularly given the many forms that RMT can take, and whether they would resemble the adaptations seen after endurance or strength (resistance) training. RMET seems promising in increasing vasomotor response (Bisconti et al., 2018), whereas respiratory muscle strength training has been shown to elicit responses in blood pressure (Vranish and Bailey, 2015; DeLucia et al., 2018), but to date no comprehensive evaluation over different vascular parameters has been performed. A new modality, where participants are required to perform very brief bouts of all-out respiratory efforts against high resistance (Wüthrich et al., 2015; Schaer et al., 2019), termed respiratory muscle sprint interval training (RMSIT), could optimize the potential responses of both traditional strength and endurance regimens. RMSIT has already been shown to be as effective as traditional RMET in improving respiratory parameters (Schaer et al., 2019), and the higher pressures could be enough to trigger more prominent vascular responses (Vranish and Bailey, 2015).

Given the lack of detailed knowledge on the long-term effects of RMT on vascular health and its potential for combination with passive vibration, the aim of the present investigation was therefore to first determine the acute effects of the different modalities on aortic stiffness and BP, and second to investigate the longitudinal effects of RMT on the cardiovascular system. We hypothesized that a protocol combining high-intensity respiratory exercises (RMSIT) combined with lower limb vibration would be able to induce larger exercise-related changes than RMET combined with vibration or than a single intervention alone. We further hypothesized that both respiratory interventions would have significant impact on indexes of cardiovascular health following a four-week training period.

## Materials and Methods

### Participants

Sixteen healthy, young individuals (8 men/8 women) took part in the acute phase of this investigation, while a separate group of 35 healthy, young individuals (17 men/18 women) took part in the longitudinal phase of this investigation (Table 1). Prior to the first experimental day participants were informed in detail about the procedures involved and gave their written informed consent. The studies were approved by the Research Ethics Committee of ETH Zurich (2014-N-03 and 2014-N-02) and conformed with the Declaration of Helsinki.

**TABLE 1:** Participants’ characteristics.

	Acute (*n* = 16)	Longitudinal		
		PLAT (*n* = 11)	RMET (*n* = 13)	RMSIT (*n* = 11)
Age (years)	27 ± 3	27 ± 6	27 ± 6	25 ± 3
Height (cm)	173 ± 10	173 ± 8	170 ± 7	173 ± 10
Weight (kg)	66 ± 14	66 ± 10	66 ± 10	66 ± 10
BMI (kg⋅m^–2^)	22 ± 3	22 ± 3	22 ± 2	22 ± 3
FVC (%pred)	108 ± 13	107 ± 9	111 ± 16	102 ± 14
FEV_1_ (%pred)	103 ± 9	102 ± 11	107 ± 12	103 ± 14
MVV_12_ (%pred)	123 ± 13	119 ± 18	119 ± 11	122 ± 18
MIP (cm H_2_O)	112 ± 28	123 ± 28	139 ± 35	129 ± 26
MIP (% pred)	122 ± 38	134 ± 30	155 ± 39	139 ± 50
MEP (cm H_2_O)	152 ± 46	181 ± 52	184 ± 41	174 ± 33
MEP (% pred)	124 ± 33	152 ± 44	157 ± 43	141 ± 37
V̇O_2__*max*_ (ml.kg^–1^.min^–1^)		51 ± 13	48 ± 14	49 ± 15

*Data are means ± SD. BMI, body mass index; FVC, forced vital capacity; % pred, percent of predicted value; FEV_1_, forced expiratory volume in 1 s; MVV_12_, maximal voluntary ventilation 12 s; MIP, maximal inspiratory pressure; MEP, maximal expiratory pressure; V̇O_2max_, maximal oxygen consumption; RMSIT, respiratory muscle sprint interval training; RMET, respiratory muscle endurance training; PLAT, placebo training.*

Sample size calculation was performed for the acute phase of the investigation, taking into consideration the short-term variability in SBP and carotid-femoral (PWV_*CF*_) (Beltrami et al., 2018) of approximately 4% and that changes of at least 5% could be expected for SBP from different exercise interventions (Naci et al., 2018), including respiratory exercises (Vranish and Bailey, 2015), while changes >10% can be expected for PWV_*CF*_ (Heffernan et al., 2007a). Assuming an α of 0.05 and power (1 – β) of 0.8, a sample size of *n* = 8 would be required to detect differences in SBP compared to a control situation (calculation performed using G^∗^Power 3.1). Eight men and 8 women were recruited so that subsequently the study would be adequately powered to investigate differences between sexes, which did not occur. The training phase of this investigation was based on a convenience sample and no formal sample size calculation took place, although there were no reasons to use different metrics as for the acute study.

### General Design

For the acute study participants visited the laboratory on six different occasions, each separated by at least 48 h and taking place at the same time of the day (±1 h). On each visit, blood pressure, PWV and heart rate variability were measured in supine position before and after six different interventions: quiet sitting (CONTROL), a 15-min session of respiratory muscle endurance exercise training (RMET), a 15-min session of respiratory muscle sprint interval training (RMSIT) plus these three variations with added vibration of the lower limbs (respectively, VIB, RMET + VIB and RMSIT + VIB). The first visit was always CONTROL, whereas the order of the additional five visits was randomized and counter-balanced. Following the measurements of the first visit lung function and respiratory muscle strength were evaluated, and participants were subsequently familiarized with the device used for the subsequent RMET and RMSIT sessions (IDIAG SpiroTiger^®^, Fehraltorf, Switzerland). This familiarization included breathing at the target ventilation and resistance level for a few minutes (RMET) and bouts (RMSIT).

For the longitudinal study, PWV and blood pressure were evaluated before and after a four-week period of either RMSIT, RMET or placebo training (PLAT). In addition, V̇O_2__max_ was measured at the start of the study, on a separate visit, using a cycling incremental test, as described elsewhere (Schaer et al., 2019). Participants were tested at the same time of day (± 1 h). A full description of the training details has already been published elsewhere (Schaer et al., 2019). Prior to the start of the training program participants were instructed on how to use the device.

Prior to each visit, participants were requested to sleep for at least 7 h on the two preceding nights, to refrain from exercise on the day before each visit, and from caffeine and alcohol intake on the day of each visit. Also, participants were instructed to have their last meal ≥ 2 h prior to each visit. Compliance with these instructions was assessed with a brief questionnaire at the start of each visit.

### Procedures and Experimental Set-up

#### Lung Function and Respiratory Muscle Strength

Lung function was measured according to current guidelines (Miller et al., 2005), using a metabolic cart with a calibrated volume sensor (Oxycon Pro; Jaeger, Höchberg, Germany). Maximal inspiratory pressure (at residual volume) and maximal expiratory pressures (at total lung capacity) were measured using a handheld device (Micro RPM; Micro Medical Ltd., Rochester, United Kingdom). Participants performed a minimum of three maneuvers, and the largest of the three highest values (differing by ≤ 5%) was selected as the maximum. Variables of pulmonary function and respiratory muscle strength are reported both as absolute values and as a percentage of predicted (Wilson et al., 1984; Stocks and Quanjer, 1995).

#### Assessment of Blood Pressure and Pulse Wave Velocity

Blood pressure and PWV were measured in supine position after 10 min of awake supine rest. Participants were lying quietly on a stretcher and were requested to keep the right arm resting parallel to the body (anatomical position), with the palms facing slightly upwards. PWV was assessed always by the same experimenter for a given participant using the Complior Analyze system (Alam Medical, Vincennes, France). Three independent piezo-electric sensors were placed on the right side of the body to simultaneously record the changes in pulse pressure at the common carotid artery, common femoral artery and radial artery. Measurements were repeated until a triplicate within 0⋅5 m⋅s^–1^ was obtained for the three segments (Wilkinson et al., 2010). Immediately prior to the start of the acquisition of PWV data, arterial blood pressure was measured in duplicate in the left arm using a commercially available device (Mio Star CP500, Zurich, Switzerland). In addition to systolic (SBP) and diastolic (DBP) blood pressure, mean arterial pressure (MAP) was calculated as 2/3 DBP + 1/3 SBP. Carotid-femoral distance was calculated by multiplying the distance between the two sensors by 0.8, as recommended by the ARTERY consensus (Wilkinson et al., 2010). The upstroke of the pulse waves was determined using the intersecting tangents method (Wilkinson et al., 2010). For the assessment of acute changes following exercise blood pressure and PWV were additionally measured 5 and 15 min following the training sessions. Participants remained on their seats for 1 min after the end of the sessions, following which they were instructed to quickly return to the stretcher. PWV was reported as carotid-femoral PWV (PWV_CF_), carotid radial PWV (PWV_CR_) and also the ratio between PWV_CF_ and PWV_CR_ (PWV_RATIO_) (Covic and Siriopol, 2015).

### Monitoring or Cardiorespiratory Parameters

Throughout the sessions, gas exchange was monitored using a calibrated metabolic cart (Oxycon pro; Jaeger, Höchberg, Germany). Cardiac function – including heart rate (HR), cardiac output (Q̇) and stroke volume (SV) – was assessed non-invasively using an impedance cardiograph (Physioflow, Manatec Biomedical, France), with electrode placement and equipment calibration following the manufacturers’ instructions. Blood pressure was continuously monitored using finger plethysmography (Nexfin, ClearSight system – Edwards Lifesciences, Irvine, California). In addition, mouth pressure was continuously measured with a pressure transducer (DPT-100, Utah Medical Products Ltd., Athlone, Republic of Ireland) connected to an amplifier (Quad Bridge Amp, ADInstruments, Bella Vista, Australia), which had its signal A/D converted (PowerLab 3516 Interface, ADInstruments, Bella Vista, Australia) and recorded on a computer using LabChart (ADInstruments, Bella Vista, Australia). The ECG channel of the Physioflow system was fed into Labchart for subsequent assessment of HRV parameters. HRV was calculated before and after the training sessions using the 5-min intervals preceding the pre-evaluation of PWV (after at least 5 min of quiet rest) and another 5-min interval before the post 15 min evaluation of PWV. The HRV module of LabChart (ADInstruments, Bella Vista, Australia) was used to detect the R-R intervals and calculate the different parameters.

### Acute Exercise Sessions

During all exercise sessions (acute study), participants sat on a chair for a total of 19 min with both feet placed (30 cm apart from each other, knee at 90° angle) on a vibration platform (Innoplate, MediCur AG, Niederrohrdorf, Switzerland). Following a 3-min monitoring of baseline metabolic state, the 15-min exercise sessions took place, and were finally followed by 1 min of quiet sitting before participants returned to the stretcher.

The RMET session consisted of 15-min continuous volitional, normocapnic hyperpnea using the SpiroTiger device (IDIAG SpiroTiger, Fehraltorf, Switzerland) at a target ventilation of 60% of the individual maximal voluntary ventilation. Tidal volume (50–60% of vital capacity) and breathing frequency (calculated accordingly) were held constant with online feedback on a screen in front of the subject. The RMSIT session consisted of 4 min of quiet sitting followed by six bouts of 60 s intense breathing using the SpiroTiger device followed by 60 s of rest (spontaneous ventilation). The diameter of the resistance was tailored with an added resistance (diameter 8.1 ± 0.8 mm) to the highest level that could be tolerated with a tidal volume equivalent to 60% of VC and a breathing frequency of 30 breaths per min. Finally, the CONTROL session consisted of quiet sitting for 15 min.

The VIB, RMET + VIB and RMSIT + VIB session followed exactly the same protocols described above, except for the fact that the platform was switched on, at a frequency that elicited the highest subjective perception of vibration for each participant (range 16–19 Hz). The peak-to-peak displacement of the platform was calculated as 5.5 mm and acceleration 2.49 g.

### Respiratory Muscle Training/Placebo-Training

The training procedures have been described in full extent elsewhere (Schaer et al., 2019). Training was performed at home and participants were required to keep a logbook of the sessions, which was used to check for training adherence and intensity. Moreover, the first and middle training session was performed under supervision in the laboratory, to ensure that participants were following the training properly.

The PLAT group trained 3-4 times a week, for 30 days using a mock asthma inhaler (HandiHaler^®^, Boehringer Ingelheim, Ingelheim, Germany) filled with lactose powder. Subjects were instructed to inhale the powder according to inhaler instructions and to then perform five full inspirations to total lung capacity using custom-made, low-resistance tubing, which elicited minimal resistance to breathing.

The RMET consisted 20 training sessions over 30 days in similar fashion to the acute investigation, except session duration was 30 min. Two consecutive days of training were followed by a day of rest, as this was shown to be effective in previous studies (Verges et al., 2007). If after 25 min of breathing subjects felt that they would not be exhausted after 30 min, f_B_ was increased by 2 breaths⋅min-^1^ and the next training session was started at this f_B_.

The RMSIT consisted of 12 training sessions, three time per week, with similar structure to the acute investigation. If respiratory exertion was ≤ 8 points after a sprint (on a 0-10 scale), subjects were instructed to increase breathing frequency (f_B_) of the next sprint by 1 breath⋅min^–1^. If subjects reached an f_B_ of 35 breaths⋅min^–1^, an orifice with a smaller diameter was inserted and f_B_ was reduced to 30 breaths⋅min^–1^.

### Data Analysis and Statistics

All data was initially checked for normality. For the acute investigation, an initial two-way repeated measures ANOVA was performed on each pair of intervention (factors vibration and time-point). As no analysis showed a significant main-effect for vibration or vibration-time interaction (data not shown) it was decided to combine the trials with and without vibration, and the averaged values were used for subsequent analysis. Changes in cardiovascular parameters during the different acute interventions were investigated using two-way repeated measures ANOVA (intervention x time-point). Where a significant effect was found, *post hoc* analysis was performed (Tukey’s multiple comparison test). Cardiorespiratory responses during the interventions were compared using one-way repeated measures ANOVA with Tukey’s *post hoc* test when required. Comparison were performed using either the final 11 minutes (equivalent duration for an RMSIT session) or the intervals equivalent to the high breathing efforts of the RMSIT sessions (six times 1-min intervals).

For the longitudinal investigation, the changes from pre to post training for blood pressure and PWV were compared between groups using a one-way ANOVA. In all cases, whenever the assumption of sphericity was violated the Geisser-Greenhouse correction was applied. Statistical significance was set at *P* < 0.050. All calculations were performed using Prism 8.0 (Graphpad, La Jolla, CA, United States).

## Results

### Respiratory Pressure and Cardiovascular Function During Respiratory Interventions

**FIGURE 1:**
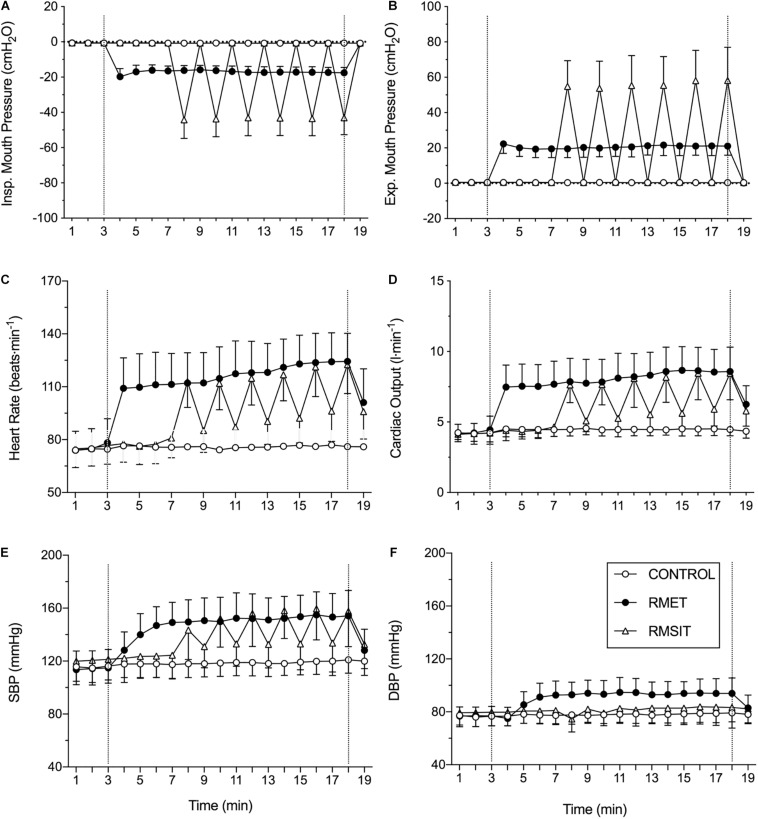
Cardiovascular responses during the different sessions. The dotted vertical lines represent the start and end of the interventions. See text for statistical differences. **(A)** inspiratory mouth pressure; **(B)** expiratory mouth pressure; **(C)** heart rate; **(D)** cardiac output; **(E)** systolic blood pressure; **(F)** diastolic blood pressure.

The pattern of mouth pressures, heart rate, cardiac output and blood pressures during the different sessions is shown in Figure 1. Over the period of equivalent duration for an RMSIT session, both inspiratory and expiratory mouth pressures were higher during RMSIT compared to RMET, which in turn was also higher than CONTROL (all *P* < 0.0001). These difference between RMSIT and RMET increased further if only the six bouts of intense ventilation are compared.

Heart rate and cardiac output were higher during the 11 min of RMET compared with RMSIT (+ 14.3 ± 6.5 bpm, *P* = 0.01; and 1.4 ± 0.7 L⋅min^–1^, *P* = 0.006), however these differences disappeared when comparing only the periods of intense ventilation (both *P* = 0.90, Figure 1). SBP was elevated during RMET and RMSIT compared to CONTROL (both *P* < 0.0001), however it was not different between the two RMT modalities (*P* = 0.266), even when comparing only the periods of intense ventilation (*P* = 0.990). DBP on the other hand was elevated during RMET compared with both RMSIT and CONTROL, regardless of whether comparing the 11 min of the RMSIT session of only the periods of intense ventilation (all *P* < 0.0001).

### Acute Effects on Cardiovascular Parameters

**FIGURE 2:**
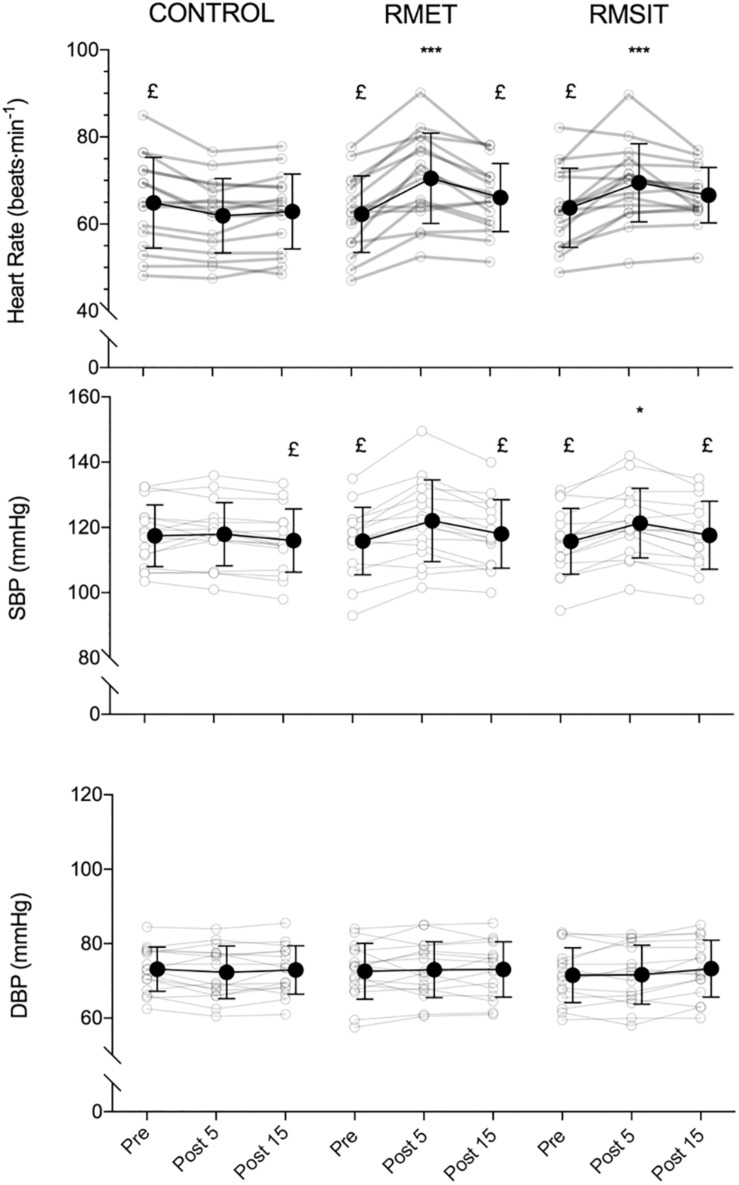
Acute responses of heart rate (top), systolic blood pressure (middle) and diastolic blood pressure (bottom) to different respiratory muscle training sessions. *p < 0.05, ***p < 0.001 vs. control at same time-point; £ *p* < 0.05 vs. post 5.

Heart rate was elevated at Post 5 following both RMET and RMSIT sessions compared with CONTROL (*P* < 0.0001) but were no longer different from Pre or the CONTROL intervention at Post 15 (Figure 2). Both RMT sessions showed increased SBP at Post 5 compared to their own Pre or Post 15 values, however only RMSIT showed a significant difference to CONTROL at Post 5 (*P* = 0.035). No within or between-intervention differences were seen for DBP.

**FIGURE 3:**
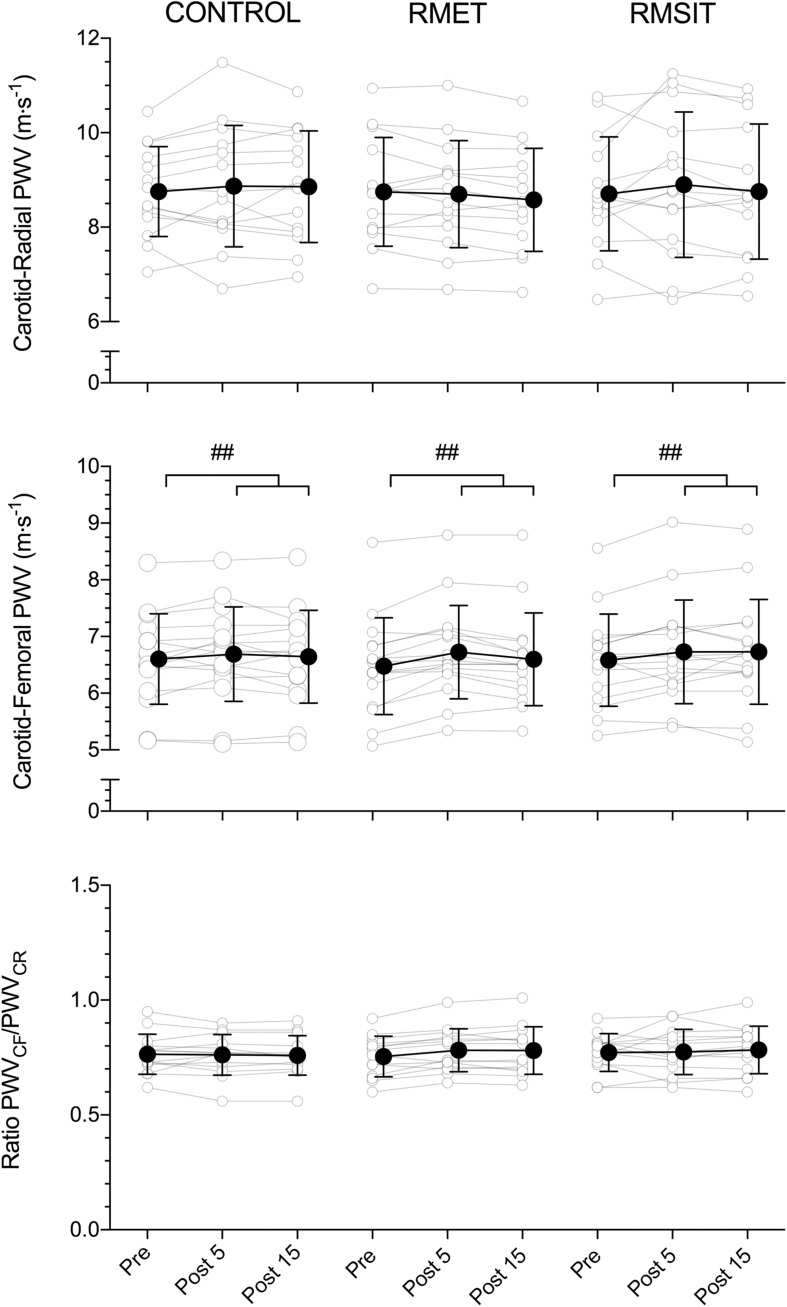
Acute responses of carotid-radial (CR) PWV (top), carotid-femoral (CF) PWV (middle) and the CF/CR ratio (bottom) to different respiratory muscle training sessions. ^##^main-effect of time for PWV_*CF*_ (middle panel), with both post 5 and post 15 significantly higher than pre (*p* < 0.01).

The effects of the different RMT sessions on PWV is shown in Figure 3. Overall, there were no effects of neither RMT modality on PWV_CR_, or PWV_RATIO_. There was a significant main effect of time for PWV_CF_ (*P* = 0.0002), with values at Pre different from both Post 5 (*P* = 0.0002) and Post 15 (*P* = 0.01). There were however no interaction effects.

Data from heart rate variability parameters is shown in Table 2. There were significant interaction effects for both SDRR (*P* = 0.0019) and VLF Power (*P* = 0.015) however *post hoc* testing was unable to locate the difference. Both RMT modalities resulted in decreased HF power post exercise (both *P* < 0.01), at which time values were lower than during CONTROL. Similarly, RMSSD decreased following both RMT sessions (*P* < 0.0001), and Post values were lower compared to CONTROL (both *P* < 0.020). There was a main-effect of time for LF/HF ratio (*P* = 0.015), although the effect was numerically stronger for the RMT sessions compared with CONTROL.

**TABLE 2:** Acute changes in indexes of HRV.

	CONTROL		RMET		RMSIT	
	**Pre**	**Post**	**Pre**	**Post**	**Pre**	**Post**
SDRR (ms)	61 ± 17	69 ± 20	71 ± 18	61 ± 15	67 ± 21	60 ± 15
VLF Power (μ s^2^)	1347 ± 789	1854 ± 1239	1461 ± 710	1601 ± 948	2099 ± 1569	1418 ± 905
LF Power μ s^2^)	1278 ± 1350	1827 ± 1653	1526 ± 1035	1184 ± 962	1196 ± 923	1045 ± 811
HF Power μ s^2^)	1276 ± 1083	1608 ± 1009	1977 ± 1633^#^	965 ± 527^∗^	1702 ± 1532	868 ± 743^*#^
LF/HF ratio	1.17 ± 0.65	1.23±0.89^*A*^	1.12 ± 0.83	1.53±0.99^*A*^	1.03 ± 0.86	1.60±0.85^*A*^
RMSSD (ms)	53 ± 20	61 ± 22	64 ± 24^#^	48 ± 16^*#^	58 ± 24	46 ± 16^*#^

*SDRR, standard deviation of the RR intervals; VLF, very low frequency; LF, low frequency; HF, high frequency; RMSSD, root mean square of the successive differences in RR intervals. ^∗^*P* < 0.05 vs. pre; ^#^*p* < 0.05 vs. control at same time-point.^*A*^main-effect for time.*

### Effects of RMT on Respiratory and Cardiovascular Parameters

Training characteristics of the different programs have been published elsewhere (Schaer et al., 2019). Briefly, all participants performed the designated 12 RMSIT sessions or 20 RMET sessions. Comparing the first to last training sessions, participants in both groups increased mean ventilation, but more so in the RMET group (group x time interaction *P* < 0.001), whereas only the RMSIT group increased the average mouth pressure during the session (group x time interaction *P* = 0.01). As a result, estimated total work of breathing improved in both groups from the first to the final training session without significant differences (∼180% increase, group x time interaction *P* = 0.143).

Both RMT modalities improved parameters of respiratory function whereas no such effects were noted following PLAT, as previously described (Schaer et al., 2019). Briefly, there was a main-effect of time for MIP (*P* = 0.017) and MVV (*P* < 0.01). Furthermore, both the RMET and RMSIT groups improved performance during an incremental breathing test, while PLAT did not.

**FIGURE 4:**
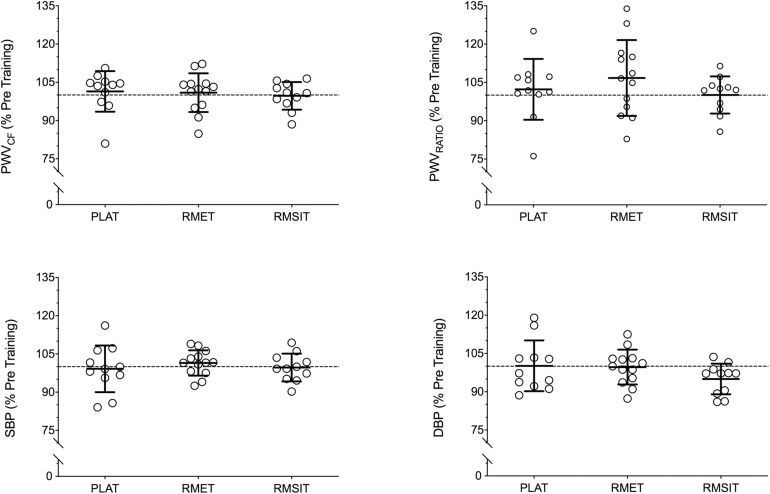
Relative changes in cardiovascular parameters following 4 weeks of respiratory muscle endurance training (RMET), respiratory muscle sprint-interval training (RMSIT), or placebo training (PLAT).

Resting BP and PWV before and after the training period are shown in Table 3. There were no differences between any of the parameters before the start of training. Following training, no changes in BP or PWV-related parameters were seen in any of the groups (Figure 4).

**TABLE 3:** Cardiovascular parameters before and after 4 weeks of RMT/placebo.

	PLAT		RMET		RMSIT	
	Pre	Post	Pre	Post	Pre	Post
SBP (mmHg)	120.2 ± 12.9	118.5 ± 9.6	118.3 ± 9.7	120.2 ± 13.2	118.8 ± 8.3	118.4 ± 9.3
DBP (mmHg)	71.8 ± 7.7	71.5 ± 6.3	72.1 ± 6.5	71.8 ± 8.1	74.9 ± 5.4	71.1 ± 6.2
MAP (mmHg)	87.9 ± 8.7	87 ± 7.1	87.5 ± 6.7	87.8 ± 9.1	89.5 ± 6.1	86.8 ± 6.5
HR (bpm)	65 ± 9	64 ± 9	64 ± 12	63 ± 13	65 ± 10	65 ± 10
PWV_*CF*_ (m⋅s^–1^)	6.7 ± 1.4	6.7 ± 1.3	6.8 ± 0.9	6.8 ± 1.1	6.3 ± 0.6	6.3 ± 0.8
PWV_*CR*_ (m⋅s^–1^)	8.5 ± 0.8	8.5 ± 0.9	9.0 ± 1.4	8.7 ± 1.5	8.2 ± 1.3	8.2 ± 1.3
PWV_*CF*_/PWV_*CR*_	0.80 ± 0.19	0.81 ± 0.18	0.74 ± 0.07	0.79 ± 0.12	0.78 ± 0.11	0.78 ± 0.10

*SBP systolic blood pressure; DBP, diastolic blood pressure; MAP: mean arterial pressure; HR, heart rate; PWV, pulse-wave velocity; CF, carotid-femoral; CR, carotid-radial.*

## Discussion

The present investigation compared the impact of different single sessions of RMT with or without lower limb vibration and the long-term impact of different RMT regimens on indexes of cardiovascular health. Although acute RMT was accompanied by cardiovascular perturbations in a range suggesting one could expect positive effects on PWV, the present results suggest that none of the interventions had significant acute effects on PWV in any arterial segment, in contrast to our initial hypothesis. Our results also indicate that either the cardiometabolic stimuli created by the different RMT programs was not sufficiently large to induce vascular adaptation or that different stimuli driving adaptative responses in opposite directions resulted in no overall change.

### Effects of Acute Cardiometabolic Stress in an RMT Session on the Cardiovascular System

In the present investigation, passive vibration of the lower limbs while in the seated position failed to elicit additional responses of BP or PWV_CF_, and for this reason trial with and without vibration were averaged together. Although vibration is believed to increase blood flow through shear stress-mediated vasodilation, thus decreasing arterial stiffness (Rittweger, 2010), these effects may be restricted to the acute changes in peripheral arteries. For example, although passive vibration of the lower limbs (10 min, 25 Hz, 2 mm amplitude, 5.4 g) acutely reduced brachial-ankle PWV in stroke patients (Koutnik et al., 2014) and healthy individuals (Wong et al., 2012) these effects were not seen for PWV_CF_, rather being a function of reduced femoral-ankle PWV.

Different exercise modalities show distinct effects on aortic stiffness: acute aerobic or endurance exercise generally leads to acute decreases in PWV (Kingwell et al., 1997; Heffernan et al., 2007a; Collier et al., 2010), while resistance exercise frequently (Heffernan et al., 2007a; Collier et al., 2010; Kingsley et al., 2016) but not always (Heffernan et al., 2007b; Okamoto et al., 2014) shows increases in aortic PWV. Acute changes in vascular stiffness have been related to different mechanisms, so that any changes in PWV likely reflect the interaction between them. Shear stress caused by increased blood flow induces vasodilation and leads to decreased arterial stiffness (Niebauer and Cooke, 1996). Increased sympathetic activation, on the other hand, leads to vasoconstriction and a concomitant increase in arterial stiffness (Katayama et al., 2015), whereas a reduction in sympathetic outflow would be expected to have the opposite effect (DeLucia et al., 2018). Finally, it has been suggested that high intra- and transmural pressures might change properties of elastin fibers on the arterial wall so that pressure is transmitted to the less distensible collagen fibers, increasing arterial stiffness (Miyachi et al., 2004; Heffernan et al., 2007b). Importantly, although this mechanism is acknowledged in the literature (Avolio et al., 1998), it is thought to take place over several years, and not after a single exercise session, when a change is stress transmission from elastin to collagen due to smooth muscle tone is more likely (O’Rourke, 1990). In the present investigation RMSSD and HF power were lower after RMT, indicating a shift in autonomic function toward increased sympathetic tonus as a possible explanation for the higher PWC_*CF*_ 5 and 15 min after exercise, even as HR and BP had returned to baseline levels.

Both RMT sessions elevated HR, cardiac output and SBP compared to CONTROL, with RMET also showing increased diastolic BP during the exercise session. Considering the RMSIT sessions as a whole, both HR and cardiac output were lower compared to RMET sessions, implying lower cardiovascular stress despite higher respiratory pressures. The average cardiac output for the RMET sessions and RMSIT bouts in the present investigation were 8.1 L⋅min^–1^ while HR ranged between 110-120 bpm, which could be considered low-intensity exercise (Fletcher et al., 2013). Also, it is in a similar range with those reported during inspiratory loading (60% MIP with 0.7 duty cycle) (St Croix et al., 2000; Welch et al., 2018). This is arguably lower than 65% V̇O_2__max_ used during aerobic exercise interventions which acutely decrease aortic PWV (Kingwell et al., 1997; Heffernan et al., 2007a).

Despite the increases in mouth pressure during the trials, changes in SBP or DBP during RMT were smaller than those seen previously during bouts of resistance training (Heffernan et al., 2007b), which also did not increase aortic stiffness (Heffernan et al., 2007b). Thus, it is even more unlikely that either session could have resulted in transient fatigue of elastin fibers, as previously suggested (Miyachi et al., 2004; Heffernan et al., 2007b). Interestingly, increased aortic PWV was seen after a 20-min set of Valsalva maneuvers aiming to produce 50 mmHg (68 cm H_2_O) of pressure. It is therefore possible that longer or even more strenuous RMT sessions could have led to a different outcome.

High carotid PWV shows a positive association with sympathetic discharge, measured by muscle sympathetic nerve activity (MSNA) (Swierblewska et al., 2010). Fatigue of the respiratory muscles increases MSNA and limb vascular resistance (Sheel et al., 2001) through the so-called respiratory muscle metaboreflex (Dempsey et al., 2008). An increase in sympathetic drive would in turn alter the sympathetic/parasympathetic balance in favor of sympathetic activity, a hypothesis also in line with the decrease in vagal activation evidenced from the changes in HF power and RMSSD after the RMT sessions. In support of this concept, the respiratory parameters during the RMT sessions in the current investigation were comparable to the ones performed by Wüthrich et al. (2015) (V̇_E_ = 59.3% MVV ± 5.0 for RMET and 64.2 ± 7.5% MVV for RMSIT; V_T_ = 3.14 l ± 0.55 for RMET and 3.10 l ± 0.54 for RMSIT), who showed that an RMSIT session has the potential to fatigue respiratory muscles similarly to an RMET session. In contrast to the somewhat convoluted findings regarding the acute effects of physical exercise on PWV, there is broader consensus that acute exercise sessions lead to a transient undershoot of SBP in the minutes following exercise, and eventually to post-exercise hypotension in the hours after exercise cessation (MacDonald, 2002), with the former effect attributed to the pooling of blood in the vascular beds of both the exercised and inactive muscles. Interestingly, this effect is noted already from 5 min of recovery after exercise with small (upper limbs) or big muscle groups (lower limbs) (MacDonald et al., 2000b), as well as after exercises of a wide range of intensities, as low as 30% of V̇O_2__max_ (Forjaz et al., 1998a) or 40% of one maximal repetition (Rezk et al., 2006). The undershoot of SBP is also present over a wide range of exercise durations (MacDonald et al., 2000a), although some evidence points toward a more prominent effect with prolonged exercise (Forjaz et al., 1998b), or higher intensities (Brito et al., 2014). In this context, it is possible that in the RMET and RMSIT the combination of low duration and intensity combined were insufficient to elicit a sufficient mismatch between the post-exercise decrease in cardiac output and vascular resistance (Halliwill, 2001).

Taken together, our data suggests that, although the cardiovascular stress of the different RMT sessions is similar to that of low-intensity aerobic exercise, changes in sympathetic activation are likely more prominent, and therefore RMT sessions lead to acute changes in PWV_*CF*_ that more closely resemble those of resistance exercise, i.e., a transient increase in aortic stiffness. At the same time, the cardiovascular strain is insufficient to induce changes in blood pressure after exercise other than a return to baseline levels.

### Effects of RMT on Variables of Cardiovascular Health

Contrary to our initial hypothesis, four weeks of either RMET or RMSIT failed to induce any changes in SBP, DBP or PWV_CF_. This finding is at odds with a recent investigation showing that six weeks of daily inspiratory muscle training (30 breaths⋅day^–1^ at 75% MIP) decreased SBP and DBP and systemic vascular resistance in healthy young adults (DeLucia et al., 2018). Another investigation by the same investigators had previously suggested that respiratory muscle training elicited decreases in both SBP and DBP provided that training resulted in large swings of thoracic pressure, regardless of whether it was caused by inspiratory or expiratory resistances (Vranish and Bailey, 2015). These opposing findings are difficult to reconcile, as the acute data in the present experiments would suggest that RMT increases aortic stiffness, not the opposite. A lower sympathetic outflow (DeLucia et al., 2018) and changes in baroreceptor sensitivity caused by repeated stimulation (Vranish and Bailey, 2015) have been suggested as a possible mechanism for the decrease in vascular resistance following IMT, but no experimental data is available to support this concept. Furthermore, Hepburn and colleagues (Hepburn et al., 2005) found that 6 weeks or 12 weeks of 30 min of hyperpnea performed 6 days per week by older adults elicited a shift in autonomic function at rest toward increased cardiac vagal tone, with increased LF:HF ratio without, however, changes in either SBP or DBP. In contrast, 8 weeks of isocapnic hyperpnea did not affect HRV parameters in young, healthy women, but nonetheless improved vascular function assessed as flow mediated dilation of the brachial artery (Bisconti et al., 2018). In this sense, it is interesting to note that RMT seems able to elicit effects in tissues that are not directly active during training, in opposition for example to knee extension, which improved vascular function of the legs but not the arms (Bisconti et al., 2019).

Conflicting findings are not uncommon regarding the effects of different exercise interventions on aortic PWV. Although aerobic exercise is seen as a tool to prevent or reduce the stiffening of the aorta that takes place with aging (Seals, 2014), and indeed several investigation are pointing toward a beneficial impact of training (Hayashi et al., 2005; Collier et al., 2008; Currie et al., 2009; Yoshizawa et al., 2009; Guimaraes et al., 2010; Greenwood et al., 2015), a more recent meta-analysis does not support this claim, except in cases where more hypertense individuals or long-term exercise programs are used (Montero et al., 2014). Likewise, the findings between aerobic or resistance training programs are equivocal. In kidney-transplant patients, both aerobic or resistance training programs decreased carotid PWV (Greenwood et al., 2015). On the other hand, 12 weeks of aerobic training, but not resistance training, decreased aortic PWV in women without changing SBP (Yoshizawa et al., 2009), while a 4-week program of either aerobic or resistance training both decreased SBP in pre-hypertensive individuals, but aerobic training decreased aortic PWV and resistance increased it (Collier et al., 2008). The reason behind these contradictions is unclear, but possibly includes methodological differences when measuring PWV, the choice of population and exercise modality and intensity, factors that will need to be investigated in more detail.

### Limitations

This investigation is not without limitations, and these should be acknowledged when interpreting our findings. The first post-exercise evaluation was performed 5 min after the end of the interventions. After pilot testing, this was defined as the minimal time taken to move the participants from the chair to the stretcher and re-position all equipment. Furthermore, the acute responses to the different sessions were evaluated only until 15-min post-exercise. This is a shorter time-span than used in other studies, however sufficient to show changes in BP and PWV (Rakobowchuk et al., 2009), especially as HR was back to baseline at 15-min post-exercise. Therefore, we are confident that changes were not missed due to the timing of the evaluations. Additionally, the vibration platform used in the current investigation was somewhat “weaker” in terms of g produced compared to other devices. However, data from literature show similar results to ours when evaluating the effect of passive vibration on aortic stiffness (Wong et al., 2012).

The RMT protocol used for RMET was different between the acute and training experiments. For the acute session, we aimed at matching sessions for duration, as intensity was already different. For that reason, we used a shorter variation of RMET that our group had already shown to significantly improve respiratory muscle endurance, the primary aim of training (Verges et al., 2008). The participants of the training experiments were part of a larger investigation, using a more established RMET protocol for the training program (Schaer et al., 2019). Even though too short a session could potentially decrease the acute effects of RMET, we note that as little as 10 min of passive limb vibration (Wong et al., 2012), or 4 min of static squatting (Figueroa et al., 2011) were enough to result in acute changes in PWV. Furthermore, the lack of changes following four weeks of training with 30 min sessions support our original conclusion from the acute study.

We kept the timing of testing constant to ± 1h, and participants were asked to refrain from eating in the 2h preceding a test. PWV can increase up to 40% following high-fat (70%) meals, but this does not hide the changes induced by exercise (Mc Clean et al., 2007). Therefore, although some variation in PWV could be due to different postprandial state, we saw no difference in PWV at baseline between the different days (Beltrami et al., 2018), and are therefore confident that the effects seen are not an artifact from postprandial variability.

Finally, our investigation included both males and females in each group, and men and women have been shown to have different PWV responses post exercise (Nieman et al., 2013). An analysis was performed treating men and women as different groups (not shown), but this did not yield any suggestion of an effect of sex on the responses to the different interventions.

### Conclusion and Outlook

Both the RMET and RMSIT interventions performed in the present investigation caused cardiometabolic perturbations in line with what could be expected from these modalities. Nonetheless, acute responses were minimal in terms of changes in PWV and BP, and went in a direction typically associated with unfavorable adaptations. The addition of passive vibration of the lower limbs had no additional effect in any of the assessed parameters.

Despite the acute changes seen, training programs using either RMET or RMSIT did not change the arterial stiffness or BP of young healthy adults. While this is reassuring, there is probably limited value for these modalities as a replacement for whole-body aerobic exercise with respect to vascular function. Importantly, healthy individuals might have little room for further improvements in vascular function, and it remains to be established whether the utilized protocols would have different effects in populations which are limited in their mobility or suffer from increased BP. In any case, RMT appears to offer no reason for concern despite the high pressures produced during the activity.

## Data Availability Statement

All study data can be made available upon request to FB, fernando.beltrami@hest.ethz.ch.

## Ethics Statement

The studies involving human participants were reviewed and approved by Ethics Committee of ETH Zurich. The patients/participants provided their written informed consent to participate in this study.

## Author Contributions

FB and CS conceptualized the experiment. DM critically reviewed the study design. FB and DM performed the assessments and wrote the initial manuscript draft. DM and FB performed the data extraction and analysis. FB, DM, and CS interpreted the results and approved the final version of this manuscript. All authors contributed to the article and approved the submitted version.

## Conflict of Interest

The authors declare that the research was conducted in the absence of any commercial or financial relationships that could be construed as a potential conflict of interest.
